# Salidroside protects endothelial cells against LPS-induced inflammatory injury by inhibiting NLRP3 and enhancing autophagy

**DOI:** 10.1186/s12906-021-03307-0

**Published:** 2021-05-19

**Authors:** Lijiao You, Di Zhang, Huan Geng, Fangyuan Sun, Ming Lei

**Affiliations:** 1 Department of Critical Care Medicine, Seventh People’s Hospital of Shanghai University of TCM, No.358 Datong Road, Gaoqiao Town, Pudong New District, Shanghai, 200137 China; 2 Department of Rehabilitation Medicine, Seventh People’s Hospital of Shanghai University of TCM, Shanghai, 200137 P.R. China

**Keywords:** Sepsis, Salidroside (SAL), Human umbilical vein endothelial cells (HUVECs), Lipopolysaccharide (LPS), Apoptosis, Inflammation, NOD-like receptor protein 3 (NLRP3), Autophagy

## Abstract

**Background:**

Salidroside (SAL) is a bioactive compound extracted from *Rhodiola rosea* with various biological properties. This study was designed to explore the functions of SAL on the endothelial damage induced by lipopolysaccharide (LPS) and its related mechanisms.

**Methods:**

Human umbilical vein endothelial cells (HUVECs) were pretreated with SAL (0, 10, 25, 50, 100 μM), and then incubated with LPS (10 μg/mL). Cell viability was evaluated by MTT assay, cell injury by lactate dehydrogenase (LDH) release, and inflammatory cytokines release by ELISA assay. Oxidative stress was evaluated by malondialdehyde (MDA) and superoxide dismutase (SOD) in cell lysate. Apoptosis was detected by flow cytometry and caspase-3 activity. Western blot were performed to determine expression levels of autophagy and NOD-like receptor protein 3 (NLRP3) related proteins.

**Results:**

SAL at 50 μM concentration showed no toxicity on HUVECs, but attenuated LPS-induced injury, as evidenced by increased cell viability, reduction in LDH level and inflammatory cytokines in culture media. SAL also reduced MDA level and increased SOD activity in HUVECs, and inhibited apoptosis rate and caspase-3 activity. (*P* < 0.05). Moreover, LPS enhanced HUVECs autophagy, and SAL pretreatment further enhanced autophagy, with increased Beclin-1 protein and decreased P62 protein. SAL also attenuated LPS-induced activation of NLRP3 inflammasome, reduced the protein expression of NLRP3-related proteins, including ASC and caspase-1. Autophagy inhibition by 3-MA markedly reversed SAL-modulated changes in cell viability and NLRP3 expression in LPS-stimulated HUVECs.

**Conclusion:**

SAL protects endothelial cells against LPS-induced injury through inhibition of NLRP3 pathways and enhancing autophagy.

## Background

Sepsis is a systemic inflammatory disease with bacteria or infectious agents, which poses a high morbidity and mortality challenge for clinicians [1]. Endothelial cells (ECs) play a major role in the systemic response to infection, therefore making them the main therapeutic targets of sepsis [2]. There are autoregulation between endothelial cells and inflammation during sepsis. Inflammatory cytokines produced from endothelial cells can help local control of infection, but systemic activation might aggravate the severity of sepsis, and lead to various complications, such as capillary permeability, hypotension, tissue hypoxia, and ultimately multiple organ dysfunction syndrome (MODS) [3].

Lipopolysaccharide (LPS) is a component of outer envelope of Gram-negative bacteria. After infection, LPS is released from outer envelope and initiates a series of inflammatory responses, often leading to apoptosis of vascular endothelial cells [4]. In cultured HUVECs, LPS treatment induced endothelial injury, with markedly reduced cell viability, and increased apoptosis, inflammatory cytokine production and oxidative stress [5]. Endothelial dysfunction can serve as a biomarker for early sepsis, and associated with some severe complications of sepsis, such as acute respiratory distress syndrome (ARSD) [6, 7]. It remains a great challenge to alleviate sepsis-induced endothelial injury. Moreover, recently some herbal medicines have been found to improve endothelial dysfunction and reduce inflammatory responses in LPS-induced HUVECs [8, 9]. Therefore, more effective drugs are needed for protection against endothelial injury in sepsis.

Traditional Chinese medicine (TCM) has shown suppressive effects on LPS-induced LPS-endothelial inflammation, including oxidative stress, cell apoptosis, microcirculatory disturbances and organ injury [10]. This protection on endothelium was validated in clinical practice on treatment of sepsis-induced acute respiratory distress syndrome [11]. Moreover, animal model showed that TCM decoction can ameliorate sepsis-induced acute lung injury, and the mechanism may be associated with decreased production of pro-inflammatory mediator by TCM decoction in LPS-pretreated HUVEC [12]. Therefore, it is urgently needed to explore roles the active component from drugs of TCM decoction in endothelial injury, so as to enhance the efficacy of decoction in sepsis-induced organ injury. *Rhodiola rosea* is a rhodiola plant of the Crassulaceae family and has shown potent protective effects on inflammatory injury for various diseases, including sepsis [13]. *Rhodiola rosea* treatment improved the survival rate of septic rats by enhancing the host’s immunity [14]. Salidroside (SAL) is an active component that is isolated from the *Rhodiola rosea* and has been known to have various activities in endothelial cells [15]. SAL also has protective effects against LPS and sepsis-induced acute lung injury in mice [16, 17]. However, whether SAL has protective effects on LPS-induced endothelial injury is unclear. Here, we designed a study to investigate the protective effects of SAL on the inflammatory injury and related mechanisms in LPS-induced endothelial injury.

## Methods

### Cell culture

HUVECs were purchased from Cell Bank of Chinese Academy of Sciences (Shanghai, China). The cells were cultured in low glucose Dulbecco modified Eagle medium (DMEM) with fatal bovine serum (FBS) at 37 °C in a 5% CO_2_ incubator. After 3 to 5 passages, HUVECs were used for experiment. HUVECs were pretreated with SAL (0–100 μM; Cat No. 43866, Sigma-Aldrich, Germany) for 2 h, and then incubated with LPS (10 μg/mL) for further 24 h.

### MTT assay

HUVECs (2 × 10^5^ cells/well) were seeded in 96-well plates containing 100 μL low glucose DMEM medium with 10% FBS, and pretreated with SAL (0–100 μM) for 2 h, followed by incubation with LPS (10 μg/mL) for 24 h. Then cells were added with MTT solution (Sigma-Aldrich, MO, USA) for 4 h incubation, and the absorbance at 570 nm was measured using a microplate reader (Bio-Rad, CA, USA). The values of each group were normalized to that of the untreated HUVECs.

### LDH assay

HUVECs (2 × 10^5^ cells/well) were seeded in 96-well plates containing 100 μL low glucose DMEM medium with 10% FBS, and pretreated with SAL (50 μM) for 2 h, and followed by incubation with LPS (10 μg/mL) for 24 h. Cytotoxicity to HUVECs was determined by LDH assay kit. Briefly, culture supernatant (100 μL) were collected from HUVECs of each sample. The samples were incubated with a LDH assay kit (Jiancheng, Nanjing, China) for 15 min at 37 °C.

### Assessment of cytokine levels

HUVECs (2 × 10^5^ cells/well) were seeded in 96-well plates containing 100 μL low glucose DMEM medium with 10% FBS, and pretreated with SAL (50 μM) for 2 h, and followed by incubation with LPS (10 μg/mL) for 24 h. Culture supernatant were collected from HUVECs after 24 h treatment of SAL and LPS. After centrifugation at 3000 g to remove cell debris, tumor necrosis factor-α (TNF-α) (DTA00D) and interleukin-1β (IL-1β) (DLB50) levels were assessed using ELISA kits (R&D Systems, Minneapolis, MN, USA).

### Determination of MDA and SOD levels

HUVECs were seeded in 2 mL media of 6-well plates at the density of 1 × 10^6^/mL, and were pretreated with SAL (50 μM) for 2 h, and followed by incubation with LPS (10 μg/mL) for 24 h. Malondialdehyde (MDA, a degraded oxidative lipid product from cell membranes) and superoxide dismutase (SOD, a ROS-scavenging enzyme) were measured at 532 nm and 560 nm using commercial assay kits (Beyotime, Nantong, China). All experiments were repeated three times independently. The MDA and SOD results are normalized to total protein (U/mg protein), and presented as nmol/mg protein and U/mg protein, respectively.

### Cell apoptosis assays

HUVECs were cultured at 1 × 10^6^/mL in 2 mL media of 6-well plates, pretreated with SAL (50 μM) for 2 h, and followed by incubation with LPS (10 μg/mL) at 37 °C for 24 h. After trypsinization, HUVECs were washed with phosphate buffered solution (PBS) three times, resuspended in 100 μL binding buffer containing 5 μL Annexin V-FITC (fluorescein isothiocyanate) and 5 μL PI (Propidium iodide), and preserved at 4 °C. Apoptosis was analyzed by flow cytometry (BD Biosciences). The data were analyzed using FlowJo software (FlowJo, Ashland, OR, USA).

### Caspase-3 activity

HUVECs were cultured and treated with SAL and LPS with the same method as apoptosis assay. Caspase-3 activity was assessed using a colorimetric caspase-3 assay kit (Cat no. C1115; Beyotime, Shanghai, China). In brief, cell lysate (30 μL) were incubated with caspase-3 substrate Ac-DEVD-pNA (10 μL) in a total volume of 100 μL. The mixture was incubated for 2 h at 37 °C and the absorbance at wavelength of 405 nm were determined. Caspase-3 activity in each group was normalized to that of control group.

### Autophagy

HUVECs were cultured on coverslips in 6-well culture plates. After pretreated with SAL (50 μM) for 2 h and incubated with LPS (10 μg/mL) for further 24 h, HUVECs were washed with PBS, fixed with 4% paraformaldehyde (pH 7.4), and blocked with 1% BSA and 0.1% Triton-X-100. Then cells were incubated with rabbit monoclonal antibody to LC3-II (ab192890; Abcam, MA, USA) at 4 °C overnight. Then cells were washed with PBS solution and incubated with FITC-linked secondary antibody for 1 h at 37 °C. After washing for three times, the cells were stained with DAPI (4′,6-diamidino-2-phenylindole) (10 mg/mL). Cells were observed under a confocal microscope (Leica, Germany). The DAPI+ and LC3-II+ cells were counted to calculate the percentage of LC3-II+ cells.

### Western blot analysis

HUVECs were frozen and homogenized in Radio Immunoprecipitation Assay (RIPA) buffer (Beyotime, China), and the total cellular protein was extracted. After quantification, protein sample (50 μg) was electrophoretically separated on a 10% SDS-PAGE gel and then the proteins were transferred to a PVDF membrane (Millipore, Billerica, MA, USA). Membranes were blocked with 5% skimmed milk and then incubated with primary antibodies against Beclin1 (1:300; ab210498), P62(1:400; ab56416), NLRP3 (1:500; ab214185), ASC (1:500; ab155970), caspase-1 (1:500; ab207802) and GAPDH (1:1000, ab9485; Abcam, MA, USA). Finally, the protein bands were visualized with a chemiluminescence detection kit (Thermo, USA).

### Statistical analysis

Data were expressed as the mean ± standard deviation (SD), were analyzed using SPSS version 20.0. One-way ANOVA was applied to compare the differences among groups, followed by Student-Newman-Keuls tests. *P* < 0.05 were considered as statistically significant.

## Results

### SAL inhibited inflammatory injury of HUVECs induced by LPS

**Fig. 1:**
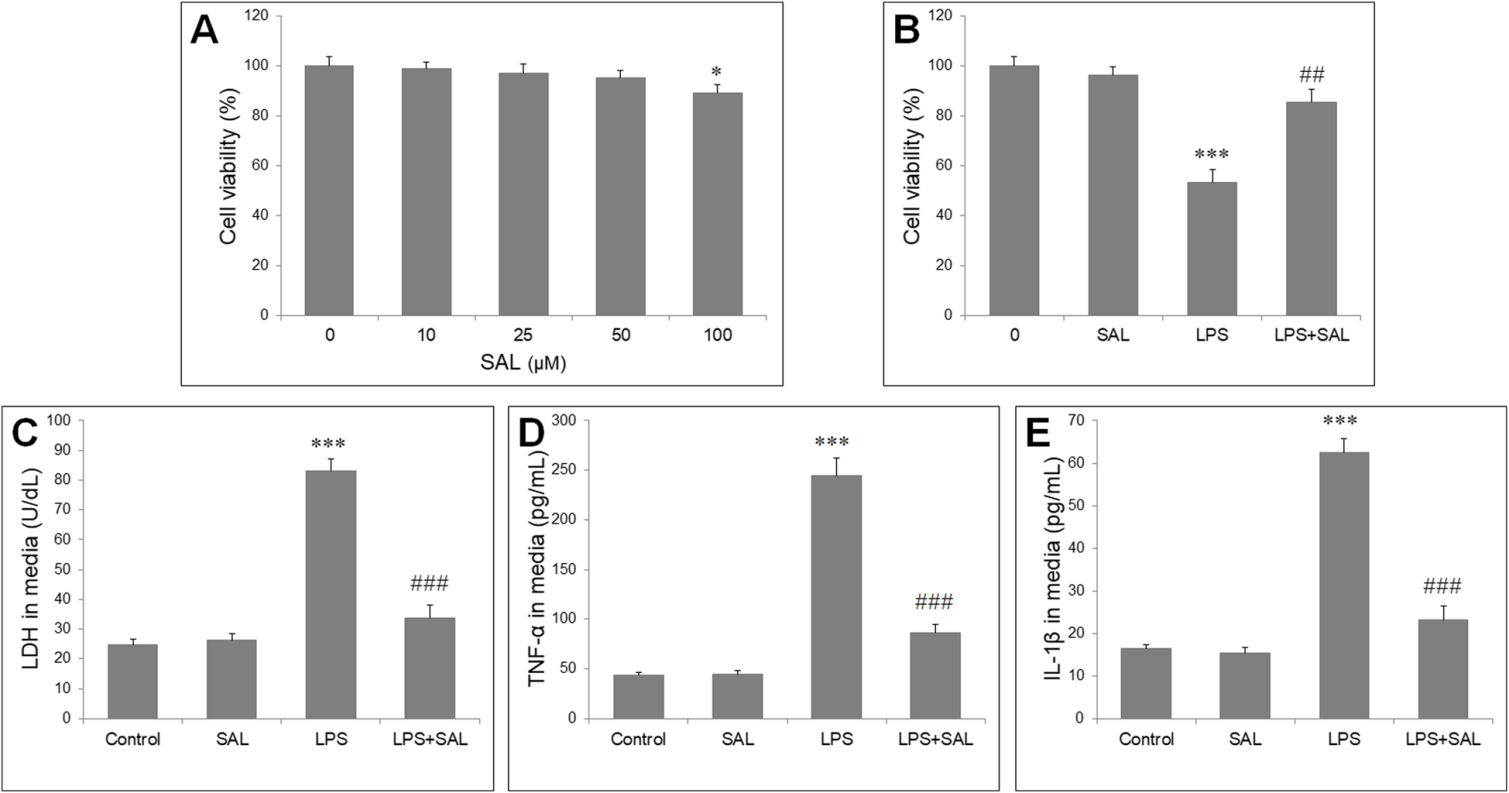
Effects of SAL on LPS-induced HUVECs injury. **a** HUVECs were treated with different concentrations of SAL (10, 25, 50, 100 μM) for 24 h. Cell viability was assessed by MTT assay. **b** HUVECs were pretreated with 50 μM SAL for 2 h, followed by incubation with LPS (10 μg/mL) for further 24 h. SAL attenuates LPS -induced reduction in cell viability. (**c**) SAL increases the release of LDH into culture media. ELISA assay was used to measure the concentrations of inflammatory cytokine in culture media. SAL attenuates the LPS-induced increase in (**d**) TNF-α and (**e**) IL-1β in culture media of HUVECs. The values presented are the means ± SD of three independent experiments, and are analyzed by ANOVA. *P < 0.05, ***P < 0.001 vs control group; ##*P* < 0.01, ###*P* < 0.001 vs LPS group

HUVECs viability was determined by MTT assay. SAL showed no cytotoxic effects on HUVECs at 10, 25 and 50 μM, and significant decreased the viability of HUVECs only at 100 μM (Fig. 1a). We chose 50 μM as the SAL concentration for following experiment. In LPS-stimulated HUVECs, SAL pretreatment markedly increased the cell viability and decreased LDH level (*P* < 0.05) (Fig. 1b, c). Furthermore, ELISA assay showed markedly increases in TNF-α and IL-1β levels in LPS-treated cells, but these promoting effects were significantly attenuated in cells with LPS and SAL (*P* < 0.05) (Fig. 1d, e).

### SAL inhibited LPS-induced oxidative stress and apoptosis of HUVECs

**Fig. 2:**
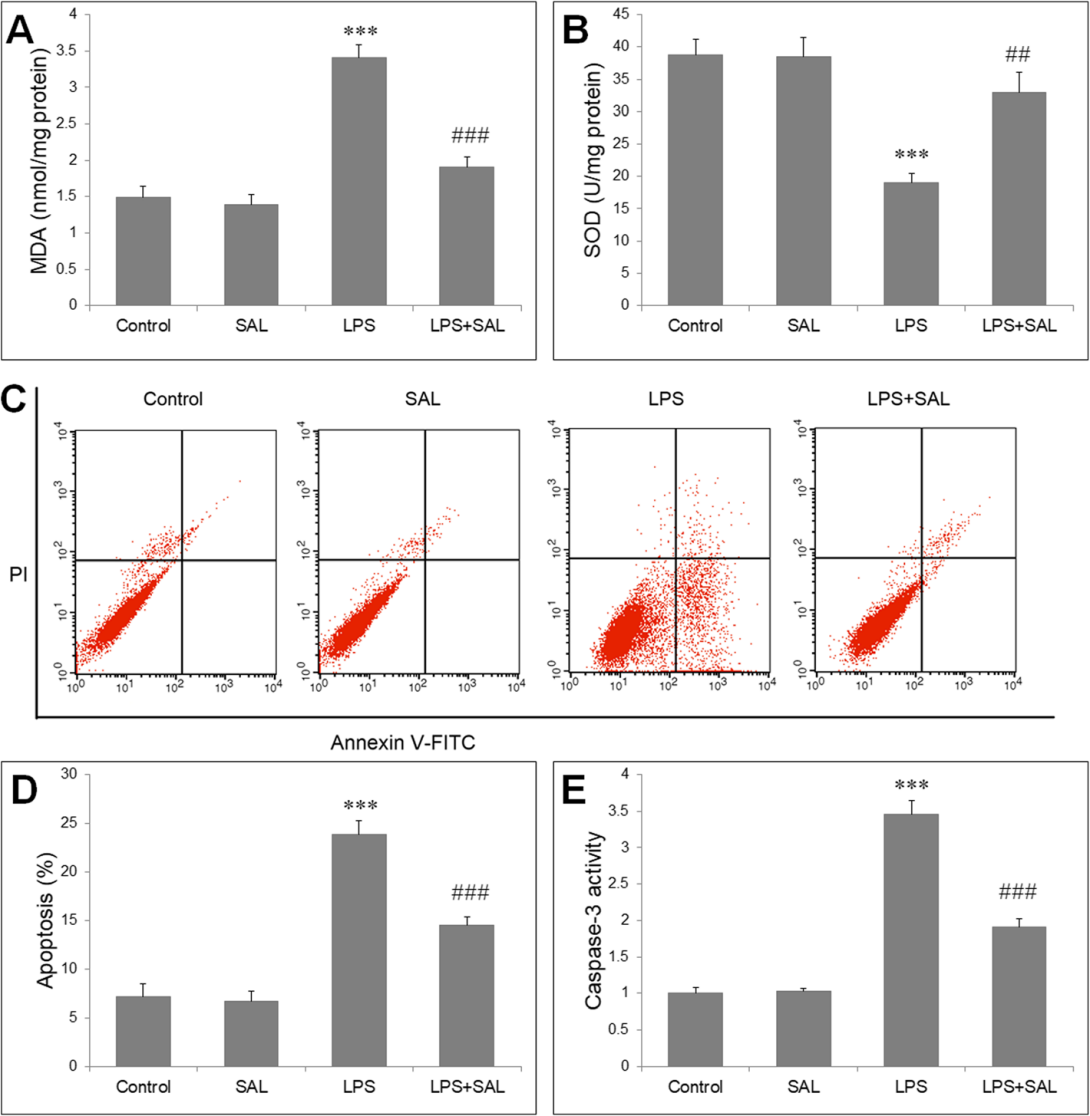
Inhibitory effects of SAL on LPS-induced HUVECs oxidative stress and apoptosis. Cells were pre-treated for 2 h with SAL (50 μM), and were exposed to LPS (10 μg/mL) for 24 h. SAL decreases (**a**) MDA level and increases (**b**) SOD activity in HUVECs exposed to LPS. Cell apoptosis was measured by Flow cytometry (**c**) Dot plot images of flow cytometry of apoptotic cells are shown. **d** Apoptotic cells analyzed by flow cytometry. **e** Caspase-3 activity relative to control cells. ***P < 0.001 vs control group; ##*P* < 0.01, ###*P* < 0.001 vs LPS group

LPS significantly increased MDA level (Fig. 2a), and decreased SOD activity (Fig. 2b) in HUVEC cells. However, the two oxidative stress indicators was reversed by SAL pretreatment (*P* < 0.05). Then HUVECs were stained with annexin V/PI to evaluate apoptosis rate. LPS significantly increased the HUVEC apoptosis rate from 7.1 to 23.9% compared with control cells (*P* < 0.05). Pre-treatment with SAL (50 μM) significantly reduced the apoptosis rate in HUVEC cells with LPS exposure (Fig. 2c, d), but SAL (50 μM) alone had no effect on the apoptosis rate. Consistently, caspase-3, a biomarker of apoptosis, was analyzed by colorimetric method. SAL attenuated the increase in caspase-3 activity induced by LPS (Fig. 2e). Taken together, LPS induced oxidative stress and apoptosis in HUVEC cells, and SAL could exert an pro-survival and antioxidant effect on LPS-induced HUVEC cells.

### SAL promoted autophagy in HUVECs with LPS

**Fig. 3:**
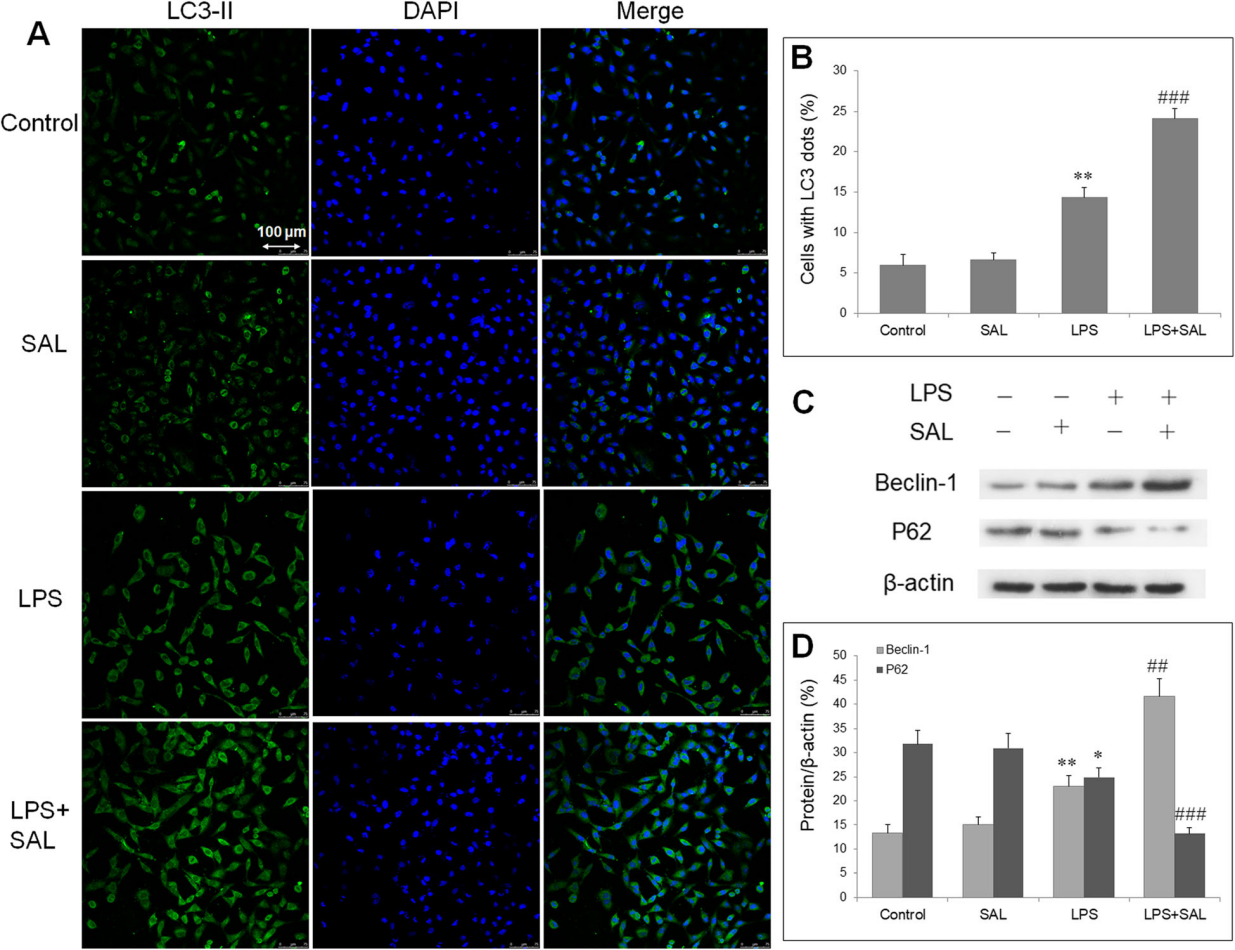
Effect of SAL on autophagy and related proteins in HUVECs. **a** HUVECs are stained with LC3-II antibody and observed under confocal microscope (magnification × 200). **b** Quantification of LC3-II+ cells. **c** Representative blots of autophagy-related proteins. **d** Regulation of SAL on protein expression of Beclin-1 and P62. *P < 0.05, **P < 0.01 vs control group; ##*P* < 0.01, ###*P* < 0.001 vs LPS group

HUVEC cells were stained with LC3-II antibody to evaluate autophagy by confocal microscopy. LPS induced autophagy in HUVECs, with increased yellow fluorescence intensity (LC3-II intensity) (Fig. 3a). SAL pretreatment further enhanced autophagy, as evidenced by markedly increased percentage LC3-II+ cells (Fig. 3b). Then western blot was performed to determine the expression of autophagy-related proteins (Fig. 3c). LPS increased Beclin1 level and decreased P62 level in HUVECs. Cotreatment with SAL further increased Beclin1 protein and decreased P62 protein (Fig. 3d,e). This indicates SAL induced autophagy in HUVECs with LPS exposure.

### SAL suppressed LPS-induced NLRP3 inflammasome activation by autophagy pathway

**Fig. 4:**
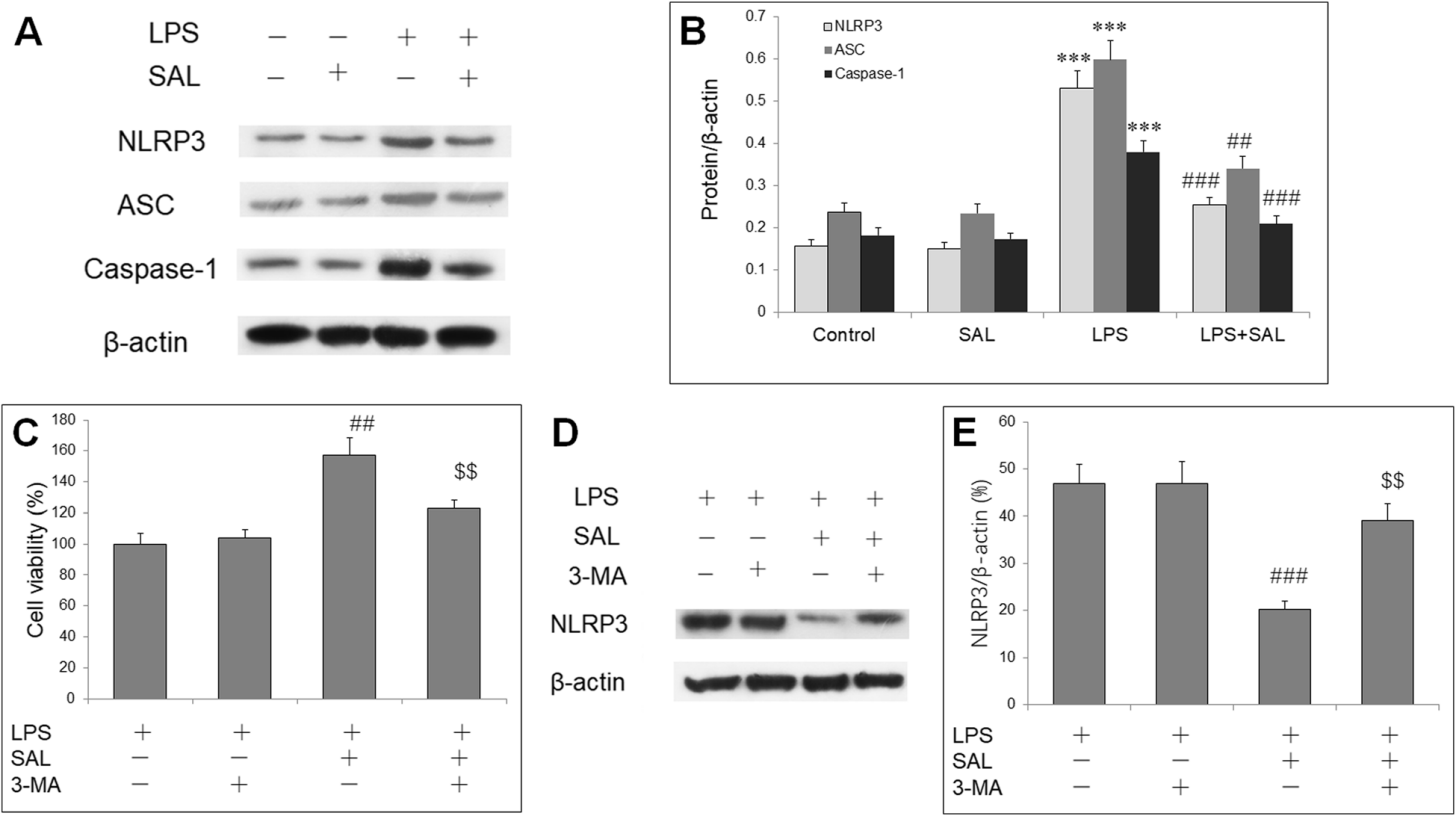
SAL suppresses NLRP3 inflammasome activation by enhancing autophagy. **a** Representative western blot bands of NLRP3 pathway proteins. **b** Quantitative results of western blot analysis on expressions of NLRP3, ASC, and caspase-1. HUVECs were pretreated with 3-MA and/or SAL, and then exposed to LPS for 24 h. **c** MTT shows 3-MA decreases cell viability in HUVECs with LPS and SAL. **d** Representative blots of NLRP3 protein by western blot analysis. **e** . 3-MA reverses SAL-induced decrease in NLRP3 protein expression. ***P < 0.001 vs control group; ##*P* < 0.01, ###*P* < 0.001 vs LPS group; $$*P* < 0.01 vs LPS + SAL group

To investigate whether NLRP3 inflammasome is involved in the protective effect of SAL in HUVECs cell injury by LPS, we determined expression of NLRP3 and its related proteins (Fig. 4a). LPS activated NLRP3 inflammasome, as evidenced by increased NLRP3, ASC and caspase-1 protein level compared with control cells, which was both reversed by SAL pretreatment (Fig. 4b). To investigate the relationship between autophagy and the protective effect of SAL, HUVECs were preincubated with SAL and a autophagy inhibitor, 3-MA and/or SAL, and then exposed to LPS for 24 h. Compared to HUVECs with LPS and SAL, 3-MA significantly decreased cell viability in HUVECs after SAL pretreatment (Fig. 4c). We then performed western blot analysis to determine then NLRP3 protein expression. 3-MA significantly reversed SAL-induced reduction in NLRP3 protein level in HUVECs with LPS exposure (Fig. 4d, e). These results suggest that SAL plays an protective effect on LPS-induced HUVECs injury by autophagy-NLRP3 pathway.

## Discussion

In this study, we investigate the protective effects of SAL on LPS-induced endothelial injury. SAL inhibited apoptosis, inflammatory cytokine production and oxidative stress in LPS-stimulated HUVECs. The protective effects of SAL were mainly associated with enhanced autophagy and suppressed NLRP3 signaling pathway. Our results provided SAL as the potential agent in sepsis-induced endothelial dysfunction.

A large number studies have shown that sepsis patients and animals suffer from endothelial injury, which is caused by excessive oxidant stress and production of inflammatory cytokines [3]. SAL reduced the production of ROS and inhibited inflammatory response, thus protected endothelial cells from injury induced by oxygen-glucose deprivation [15]. Moreover, SAL has wide protective effects on endothelial injury induced by various conditions, such as high glucose, advanced glycation end product (AGE), and oxidized low-density lipoprotein (ox-LDL) [18–20]. Considering the key role of endothelial cells in initiation and progression of sepsis, we hypothesized that SAL can also demonstrate protective effect on LPS-induced endothelial cells, which is a well in vitro model of septic endothelial injury. Therefore, we applied SAL to HUVECs with LPS stimulation, and showed SAL is a potential agent for endothelial protection. The mechanisms of SAL may be associated with induction of autophagy.

Our study shows that SAL enhanced autophagy in LPS-induced HUVECs, which was further supported by regulation of autophagy-related proteins, Beclin-1 and P62. Autophagy is a degradation process in which damaged organelles are delivered to lysosome degradation, especially under the conditions of oxidation stress and inflammatory response. Autophagy plays an contradictory role in cell survival after sepsis. Autophagy mediated the cytokine storm and vascular leakage in sepsis [21]. However, autophagy has been reported to mediate the protective effect on LPS-induced cardiac dysfunction by modulating inflammatory and oxidative stress [22]. This can be explained by the dual role of autophagy in modulating cell survival: excessive autophagy can cause cell death, it is primarily a process that promotes survival under various stress conditions through degradation and clearance of intracellular damaged organelles [23]. This indicates that SAL induced autophagy is protective in LPS-induced cardiac dysfunction. Considering the common origin of cardiomyocytes and endothelial cells, we hypothesized that SAL can also demonstrate protective effect on LPS-induced endothelial cells. This hypothesis is also supported by our study that SAL-induced increase in cell viability of HUVECs was reversed by specific autophagy inhibitor 3-MA. LPS itself can induce autophagy in cultured endothelial cells, but whether this autophagy promote cell death or survival remains unclear and depends on different conditions and stimulant [24]. In LPS-induced acute lung injury mice, enhanced autophagy of pulmonary endothelial cells promoted cell survival, thus significantly reduced the severity of lung injury [25]. In our study, SAL-induced increase in HUVECs viability was dependent on autophagy induction, this implies that autophagy might be also associated with oxidative damage and apoptosis of HUVECs. A report showed that in HUVECs with hydrogen peroxide (H_2_O_2_) exposure, SAL exerted cytoprotective effects against oxidative injury, and induced autophagy simultaneously. This autophagy was essential for anti-oxidative and survival of HUVECs, as inhibition of autophagy by 3-MA further promoted HUVECs apoptosis [26]. This indicates that SAL also can induce protective autophagy in HUVECs with LPS exposure in our study. In fact, SAL also induce autophagy in HUVECs with ox-LDL exposure [27]. Our study provides SAL as a potential agent to prevent endothelial oxidative injury induced by sepsis, and autophagy is a key therapeutic target.

Our study shows that SAL inhibited NLRP3 inflammasome pathway in LPS-induced HUVECs, with reduced expression of NLRP3, ASC and caspase-1. NLRP3 inflammasome is a mediator in the innate immune system, and includes three main members, namely NLRP3, ASC and caspase-1. After activation, NLRP3 inflammasome activates caspase-1, promotes production of inflammatory mediators and initiates inflammation [28]. This was validated by increased protein expression of NLRP3, ASC and caspase-1, and supernatant TNF-α IL-1β in HUVECs after LPS stimulation. Activation of the NLRP3 inflammasome promoted endothelial dysfunction of early sepsis in mice [29]. Therefore, NLRP3 inflammasome is regarded as a therapeutic target of septic endothelial dysfunction and its inhibition mediates the cytoprotective effects in LPS-induced HUVECs injury by Procyanidin B2 [30]. Our study added SAL as another agent that targets NLRP3 inflammasome in protection on endothelial damage, which is supported by a recent report that SAL suppresses caspase-1 activation and IL-1β release in LPS-induced HUVECs [31]. Moreover, inhibition of NLRP3 by SAL is dependent on autophagy activation, as this change can be reversed by an autophagy inhibitor 3-MA in HUVECs with LPS exposure. Autophagy is closely associated with NLRP3 inflammasome activation. Under the condition of excessive inflammatory responses, autophagy controls the detrimental inflammation through inflammasome inactivation [32], thus contributes to the degradation of NLRP3 and reduction of IL-1β release [33]. Autophagy activation demonstrated protective effect on sepsis-induced organ damage through NLRP3 inflammasome inactivation [34]. A recent report showed that autophagy can suppress NLRP3 inflammasome by eliminate intracellular ROS, thus making a autophagy-ROS-NLRP3 axis for protection against ischemic myocardial injury [35]. It can be speculated that in this study, SAL-induced autophagy might also suppress oxidative stress, thus lead to NLRP3 inhibition. Taken together, our study propose autophagy-NLRP3 axis is a new pathway in endothelial injury of sepsis, which deserves further investigation.

## Conclusions

SAL alleviates LPS-induced HUVECs injury by activation of autophagy and inhibition of NLRP3 pathway. SAL might be used as a potential agent for sepsis-induced endothelial dysfunction.

## Data Availability

The data in support of the results are available from the corresponding author on reasonable request.

## Abbreviations

AGE: Advanced glycation end product
HUVECs: Human umbilical vein endothelial cells
IL-1β: Interleukin-1β
LDH: Lactate dehydrogenase
LPS: Lipopolysaccharide
MDA: Malondialdehyde
NLRP3: NOD-like receptor protein 3
ox-LDL: Oxidized low-density lipoprotein
SAL: Salidroside
SOD: Superoxide dismutase
TNF-α: Tumor necrosis factor-α
